# Metal deposited nanoparticles as “bridge materials” for lead-free solder nanocomposites

**DOI:** 10.1007/s13204-023-02898-z

**Published:** 2023-07-07

**Authors:** Yuriy Plevachuk, Peter Švec, Peter Švec, Lubomir Orovcik, Otto Bajana, Andriy Yakymovych, Alexander Rud

**Affiliations:** 1 Department of Metal Physics, Ivan Franko National University of Lviv, Kyrylo and Mefodiy Str. 8, Lviv, 79005 Ukraine; 2 Institute of Physics, Slovak Academy of Sciences, Dubravska Cesta 9, 84511 Bratislava, Slovakia; 3 Centre of Excellence for Advanced Materials Application, Slovak Academy of Sciences, Dubravska Cesta 9, 84511 Bratislava, Slovakia; 4 Institute of Materials and Machine Mechanics, Slovak Academy of Sciences, Dubravska Cesta 9, 84513 Bratislava, Slovakia; 5 Institute of Chemical Technologies and Analytics, TU Wien, Getreidemarkt 9/164, 1060 Vienna, Austria; 6 G.V. Kurdyumov Institute for Metal Physics of NAS of Ukraine, Academician Vernadsky Boulevard36, Kyiv, 03142 Ukraine

**Keywords:** Lead-free solders, Carbon nanoparticles, Solder joint, Microstructure, Shear strength

## Abstract

An influence of carbon nanotubes and carbon nanospheres coated by Au–Pd and Pt on the microstructure of solder/copper joints at room temperature and after aging at sub-zero temperature. The carbon nanosized admixtures were mixed with ternary Sn3.0Ag0.5Cu matrix to prepare a composite solder. The microstructure of the solder joints between the nanocomposite solders and a copper substrate was studied by scanning electron microscopy. It was found that minor (0.05 wt. %) admixtures of both the carbon nanospheres and nanotubes increase the shear strength of the solder joints and reduce the growth rate of the intermetallic Cu_6_Sn_5_ layer, formed at the interface between solder and copper. This effect may be related to the adsorption of nanoinclusions on the grain surface during the solidification process. Comparative analysis suggests that exposure for 2 months at 253 K does not lead to deterioration of such an important mechanical characteristic of the solder joint as shear strength, indicating the possibility of using these nanocomposite solders in microelectronic equipment even at temperatures below 0 ℃.

## Introduction

The intensive development of the electronic industry and the related requirements for the miniaturization of solder joints require the improvement of the mechanical properties of lead-free solders (LFS), in particular, the strength and reliability of the joints. The three leading commercial ternary alloys Sn–Ag–Cu (SAC) of near-eutectic or eutectic compositions, known as SAC305, SAC387 and SAC405, are considered the most promising in this regard and are frequently used as materials for lead-free solders in microelectronics (Cheng et al. 2017; Tsao 2011a, b; Gain et al. 2011a, b, c; Haseeb et al. 2012). The demand for such materials with improved stability and reliability is constantly increasing. In this regard, it is important to improve various mechanical and physical properties, such as creep and fatigue resistance, yield strength, thermal resistance, as well as electrical characteristics. Reliability of soldered joints depends on intermetallic compounds (IMC), which is formed at the boundary of such joints. In the case of a thin IMC layer, the wettability between the solder and the substrate is better, while further growth of the IMC can lead to a weakening of the joints, since intermetallic compounds are known to be brittle. Brittle fractures are caused by different temperature conditions during the processes of thermal cycling and thermal aging, due to different coefficients of thermal expansion between the connecting components and the substrates. However, unlike traditional lead–tin solders, SAC solders generally have higher melting points and higher tin content. Therefore, the formation and further growth of the IMC layer occurs faster in the SAC solder joints, which leads to brittle fractures and a decrease in the service life of the joint due to thermal fatigue (Cheng et al. 2009; Wu et al. 2004; Marques et al. 2013; Li et al. 2012).

To improve the properties listed above, various impurities were added to the SAC solder alloy, which strengthen the basic matrix (see Li et al. 2021 and references therein). It was revealed that nanosized impurities have even more positive effect on increasing the strength of solder joints. This statement was also confirmed by our previous studies, devoted to the influence of nanosized metal Co (Yakymovych et al. 2017a, b), Co-Pd (Yakymovych et al. 2020), Ni (Yakymovych et al. 2017a), Ni–Sn (Yakymovych et al. 2018; Yakymovych et al. 2022) as well as ceramic Al_2_O_3_, SiO_2_, TiO_2_, and ZrO_2_ (Yakymovych et al. 2016; Aspalter et al. 2020) admixtures on physical properties and the microstructure of Sn–Ag–Cu-based solders. Since shear strength is one of the key mechanical characteristics that ensure the reliability of soldered joints, its improvement is of particular interest and importance. In view of this, the addition of namely nanosized ceramic particles to the solder matrix to form composites is a cost-effective approach to improve the shear strength, as shown by the example of ZrO_2_, CeO (Li et al. 2019) or ZnO (Fawzy et al. 2013) admixtures to the SAC matrix.

Among other nanoparticles, carbon nanotubes (CNTs) occupy a prominent place as a reinforcing material. Studies have shown that composite solders with CNTs have better mechanical properties than solders without them. Composite solders exhibit also a lower diffusion coefficient that limits the IMC growth and increases the mechanical strength of solder joints. It was revealed that the dispersion and homogeneous mixing between multi-walled carbon nanotubes and the Sn_95.8_Ag_3.5_Cu_0.7_ matrix can be achieved by processes of powder metallurgy (Nai et al. 2008, 2009). Important data about the melting temperatures of hardened alloys were reported in (Kumar et al. 2008).

Besides the filamentous morphology of graphene conducting to CNT, carbon can bond in other different ways to create structures with dissimilar properties. The pairing of pentagonal and heptagonal carbon rings can result in the formation of carbon nanospheres (CNS) (Nieto-Marquez et al. 2011; Rud et al. 2023). This nanostructure attracted recently significant research activity. In its spherical arrangement, the graphite sheets are not closed shells but rather waving flakes that follow the curvature of the sphere, creating many open edges at the surface. Contrary to the chemically inert fullerene, the unclosed graphitic flakes provide reactive dangling bonds that can enhance surface reactions, suggesting CNS as good candidates for catalytic, adsorption and other applications, as well as for the solder alloys strengthening.

One of the problems arising during mixing carbon nanoparticles with the solder is that they are non-wettable by metal melts, so it is very difficult to achieve their homogeneous distribution in the melt, as well as to avoid pushing them out of the molten solder in course of the reflowing. To solve this problem, metallic coatings can be applied to the surface of carbon materials to improve adaptation to the solder matrices before implementation. For example, Ni atoms were deposited on the surface of ceramic reinforcing additions, thus forming core–shell structures (Kumar et al. 2020, 2021). The metal-coated layer formed in this way formed a strong “bridge” that reacted with the lead-free solder matrix to form an intermetallic layer in course of soldering (Han et al. 2012; Chen et al. 2022). A similar positive effect was recently discovered when CNTs were deposited with Au nanosized particles (Plevachuk et al. 2019).

Another important requirement for the LFS application is their reliability in a wide range of operating temperatures, in particular at low temperatures, because for, e.g., application in aerospace conditions, it is important to know the characteristics of the mechanical properties behavior of components during operation in extreme thermodynamic conditions (Wu et al. 2021; Li et al. 2020; Lupinacci et al. 2013; Li et al. 2017). The use of such solders in microelectronic devices at cryogenic temperatures may not be suitable, as Sn-based solders can become brittle due to the allotropic phase transformation of tin, known as “tin pest”.

However, along with the cryogenic temperature range, ensuring the efficient functioning of electronic devices is also important at sub-zero temperatures in terrestrial conditions. Studies of the thermocycling effect at elevated temperatures were carried out (Tan et al. 2015; Tian et al. 2018), but properties behavior of the solder joints in the sub-zero temperature range has not been studied sufficiently. In this work, the influence of multi-walled carbon nanotubes and carbon nanospheres coated with Au–Pd and Pt nanoparticles on the properties of SAC305 solder both at room temperature and after aging for 2 months at 253 K (minus 20 °C), was investigated.

The compositions of the investigated alloys and their designations in the text are given below.

## Methods/experimental

Ternary alloy Sn_96.5_Ag_3_Cu_0.5_ (in weight percent) (SAC305) was used as the metal matrix. The SAC305 basic material was employed in a form of a NP505-HR paste from Kester company, which is a zero-halogen solder paste formula for high reliability applications.

**Fig. 1:**
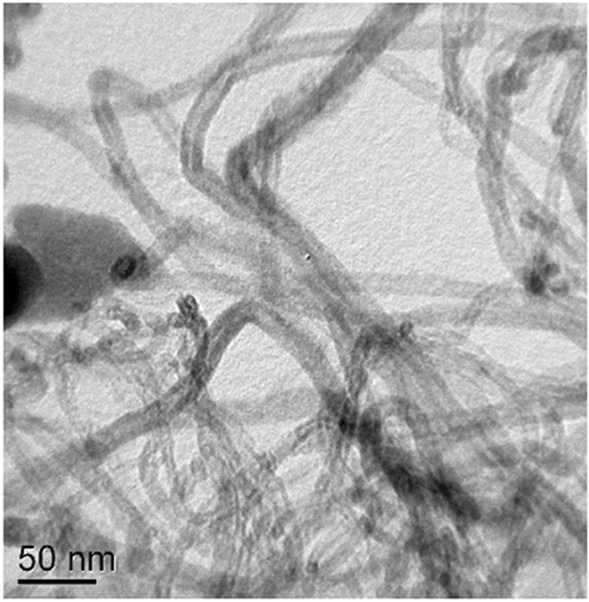
Carbon nanotubes (TEM)

Two types of nanosized admixtures, namely, carbon nanotubes (CNTs) and carbon nanospheres (CNSs) were mixed with the basic SAC305 alloy. Carbon spheres were chosen additionally due to the latest data and ideas suggesting that the influence of such admixtures on physical properties of SAC matrix would be also effective. Multi-walled carbon nanotubes with the diameter of about 15–20 nm were synthesized by the method of catalytic pyrolysis (CCVD), using an iron-containing catalyst obtained by coprecipitation of hydroxides of aluminum, magnesium, and ferric iron (Kartel et al. 2016) (Fig. 1).

**Fig. 2:**
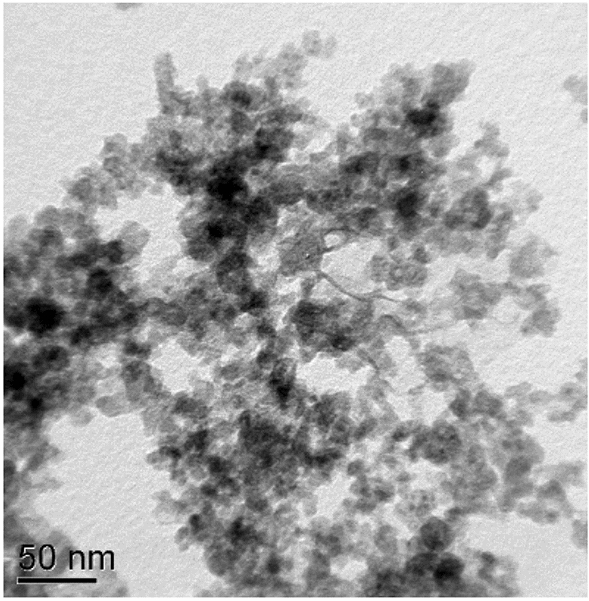
Carbon nanospheres (TEM)

The electrical discharge methods, based on different principles, namely, the structural phase transformations of carbon that occur as a result of an electrical explosion of graphite, and the low energy electrical discharge action on the molecules of organic liquids, were used for obtaining carbon nanospheres in benzene (CNS1), propane-butane mixture (CNS2) and cyclohexane (CNS3) (Rud et al. 2012) (Fig. 2).

To cover CNT and CNS with Au–Pd and Pt metal vapor precipitates, a high-resolution Gatan ion beam coater (Model 681) was used. The ion beams were generated at ~ 8 keV/300 mA. The system was co-pumped using a liquid nitrogen cold trap placed above the molecular drag pump to further improve the system base pressure.

Since the volume density of CNTs is in the range of 20–40 g/dm^3^ that is 3 orders of magnitude less than the density of their walls (approx. 2.2 g/cm^3^), nanotubes are extremely light, and therefore, accordingly, have a rather large volume container. Based on analysis of former studies (Plevachuk et al. 2019) the amount of 0.05 wt.% CNTs and CNSs has been added to the SAC305 matrix in present experiments.

For soldered joints, 3 mm thick copper disks with a diameter of 10 and 15 mm were used as substrates. Before applying the nanocomposite solder, we grounded and polished the Cu plates with Al_2_O_3_ powders, 1 μm and 0.3 μm thick. Samples were cleaned with ethanol and immersed in a 10 vol.% sulfuric acid solution for 2 min to remove the oxide layer arising from the topmost surface. Two copper disks with solder inside were placed in an electric resistance furnace, heated to 523 K and held at this temperature for 300 s. After cooling the solder joints were cleaned of flux residues (Yakymovych et al. 2020). The hardened sample was cut, and its cross-section was carefully prepared for metallographic analysis. The microstructure of the solder joints was investigated by scanning electron microscopy (SEM). JEOL JSM-7600F and JEOL JSM-6610 equipped with an energy dispersive x-ray (EDX) analyzer [Oxford Instruments (OI), X-max 50 mm^2^], were used. The COMPO (backscattered electron imaging) and the SEI (secondary electron imaging) modes were used for surface observation. Analysis of the sizes of the intermetallic layers formed between the solder and the copper discs was performed using the Digimizer software. Due to the unevenness of the IMC layers along the interface, an average value of IMC thickness (d) was determined from the following equation:

1 $$d \, = \, S/L,$$

where *S* is the area of IMC layer obtained from the micrograph and *L* is the length of the IMC along the interface. The accuracy of determining the size of the intermetallic layers was ensured by reducing the main sources of Gaussian noise in the digital images using traditional spatial filtering methods to remove noise, such as mean nvolution) filtering, median filtering and Gaussian smoothing, which are available in the Digimizer software.

**Fig. 3:**
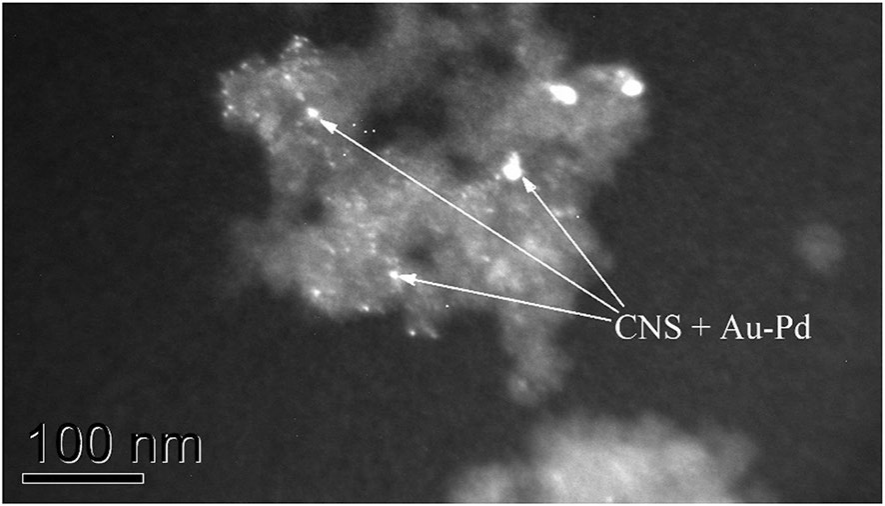
Carbon nanospheres coated by Au–Pd (TEM)

Morphology of the coated by metals and un-coated CNTs and CNSs were analyzed by transmission electron microscopy (TEM) with a conventional JEOL JEM-2000FX electron microscope operated at 200 kV. The micrographs of the samples have been taken in bright as well as in dark field imaging mode. A typical TEM micrograph of a Au–Pd-coated carbon nanospheres is presented in Fig. 3.

The shear strength of the solder/substrate joints was determined by a push-off method with a loading rate of 1 × 10^–3^ m·min^−1^ (Zwick/Roell Z 100).

## Results and discussion

**Fig. 4:**
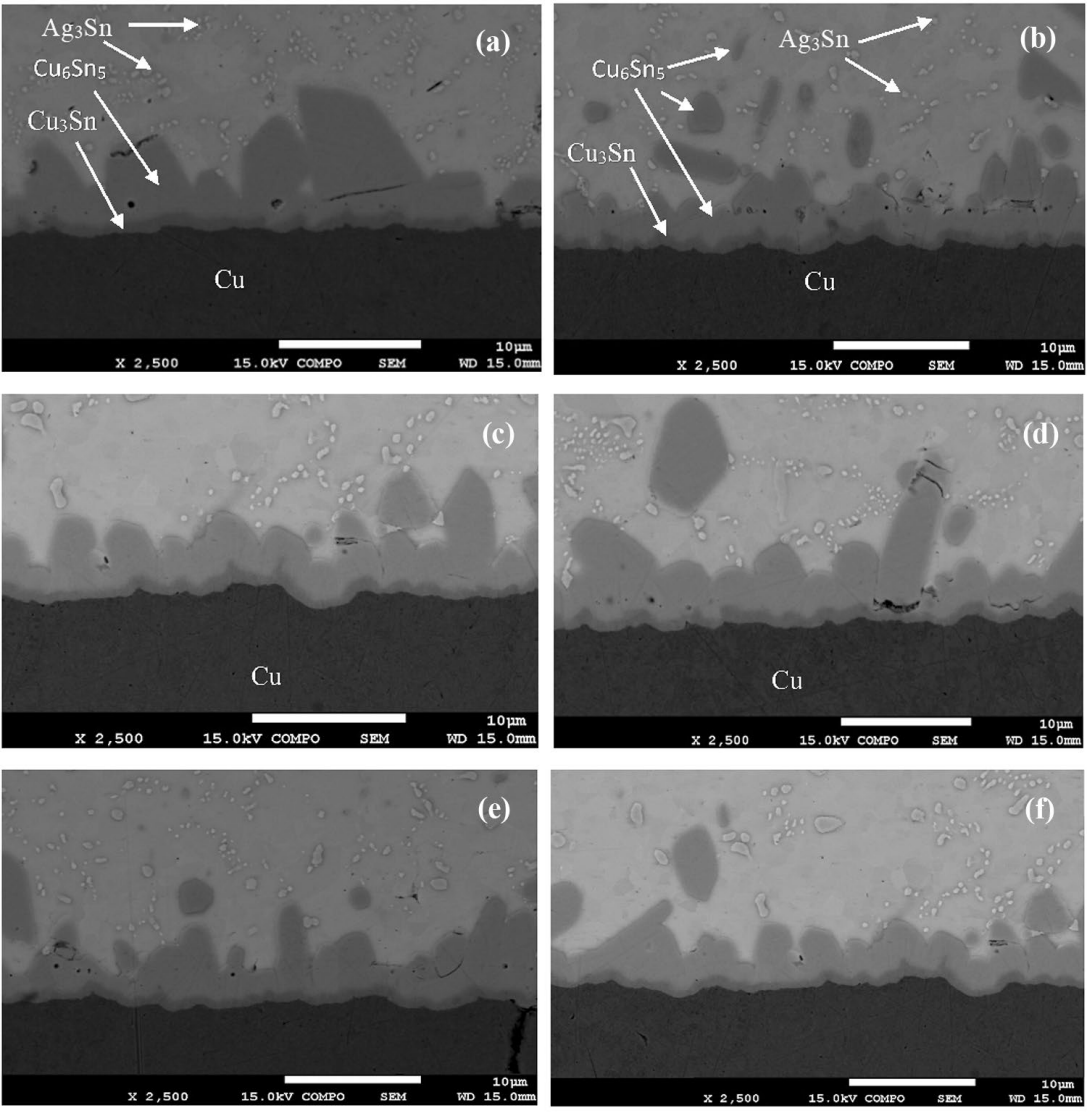
SEM micrographs of as-solidified SAC305 (**a**) and SAC305 + CNS1 + AuPd (**b**); SAC305 + CNS2 + AuPd (**c**); SAC305 + CNS3 + AuPd (**d**); SAC305 + CNT + AuPd (**e**); 6-SAC305 + CNT + Pt (**f**)

Cross-sectional micrographs of the as-solidified joints between SAC305 nanocomposite solders with carbon nanoparticles coated by Au–Pd, Pt and copper substrate are presented in Fig. 4. As can be seen, two intermetallic layers are formed between the bulk solder and the copper substrate, namely, a thinner Cu_3_Sn layer in direct contact with the substrate, and a thicker Cu_6_Sn_5_ layer above it, in contact with the bulk melt. The bulk solder consists of Ag_3_Sn and Cu_6_Sn_5_ particles surrounded by a nearly pure Sn matrix. A small amount of Ag has no influence on the Sn-Cu reaction, and the interfacial phases are similar to those in the pure Sn-Cu couples. In the binary Cu-Sn system, several peritectic reactions occur, resulting in formation of several intermetallic phases. It is known from previous studies that the ε-Cu_3_Sn phase as well as the $\eta^{\prime}$-Cu_6_Sn_5_ phase can exist at the room temperature for the alloy joint enriched with Cu and Sn (see Hwang et al. 2003 and references therein).

The Cu_6_Sn_5_ intermetallic compound formed typical scallop-type grains at the SAC305/Cu_3_Sn interface extending into the solder matrix (Fig. 4a). This intermetallic layer of a scallop-type shape is caused by a dissolution of copper atoms from the substrate into the molten solder until the solder becomes supersaturated with Cu (Laurila et al. 2005). The black dots in the IMC layer are Kirkendall voids formed in the Cu_3_Sn/Cu_6_Sn_5_ and Cu/Cu_3_Sn interfaces.

The IMC phases were observed also in the bulk solder (Fig. 4a). Along with rather large Cu_6_Sn_5_ grains small Ag_3_Sn formations also appeared in the bulk matrix but are not visible in the SAC solder/Cu as they are partly adsorbed on the Cu_6_Sn_5_ surface (Tsao 2011a, b).

Admixtures of the Au–Pd coated CNS to the SAC305 matrix led to some transformation of the solder joint layers where the shape of the scallop-type cusps becomes more rounded, while the thin Cu_3_Sn layer that touches the Cu substrate maintains a planar shape (Fig. 4b–d). The same trend was observed in the solder joints between SAC305 containing CNTs coated by Au–Pd or Pt nanoparticles and a copper substrate (Fig. 4e–d). These trends can be explained by similar morphology and the fact that they both consist of sp2 hybridized graphene shells with an interlayer spacing close to that characteristic of graphite. This similarity led to an equally uniform distribution of sputtered metal atoms on the surfaces of both kinds of nanoparticles (See Table 1).

**Table 1:** The compositions of the investigated alloys and their designations in the text

Alloy composition, wt. %	Designations in the text
Sn3.5Ag0.5Cu	SAC305
Sn3.5Ag0.5Cu-0.05(CNS1 + AuPd)	SAC305 + CNS1 + AuPd
Sn3.5Ag0.5Cu-0.05(CNS2 + AuPd)	SAC305 + CNS2 + AuPd
Sn3.5Ag0.5Cu-0.05(CNS3 + AuPd)	SAC305 + CNS3 + AuPd
Sn3.5Ag0.5Cu-0.05(CNT + AuPd)	SAC305 + CNT + AuPd
Sn3.5Ag0.5Cu-0.05(CNT + Pt)	SAC305 + CNT + Pt

**Fig. 5:**
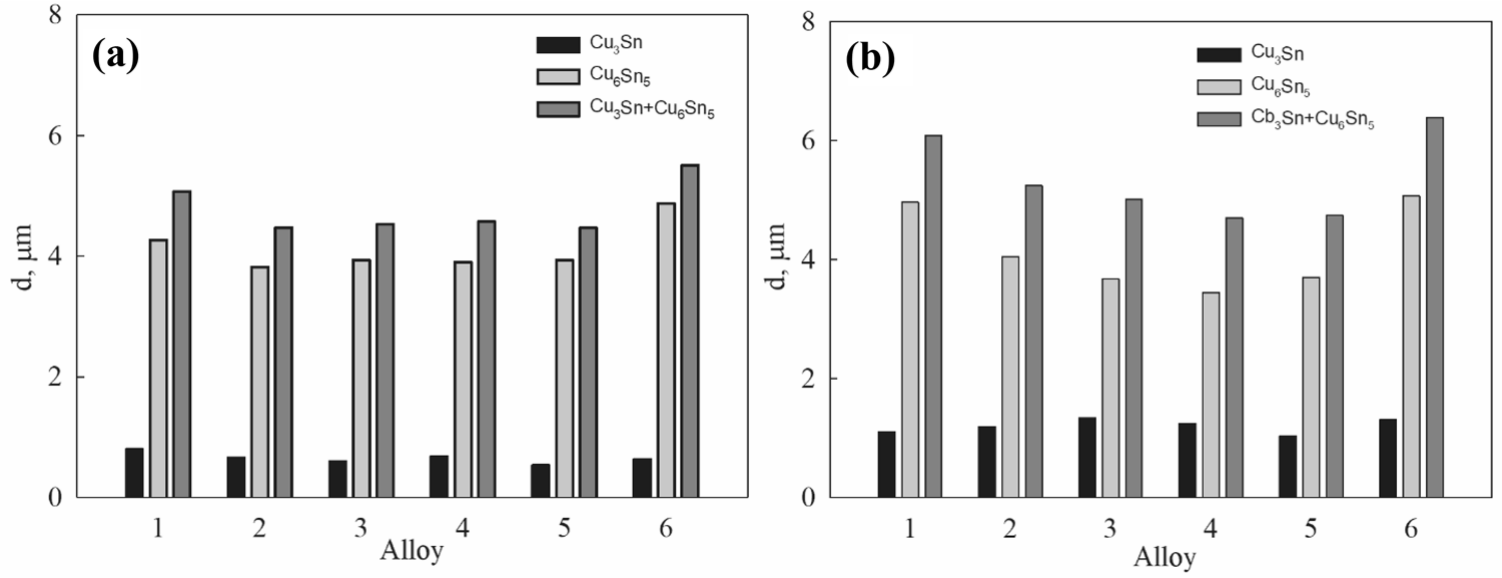
Average thickness of the interfacial Cu_3_Sn and Cu_6_Sn_5_ IMCs layers formed at the as-reflow Cu/SAC305 + nanoadmixtures/Cu at 293 K (**a**) and after aging for 2 months at 253 K (**b**) 1–SAC305; 2–SAC305 + CNS1 + AuPd; 3–SAC305 + CNS2 + AuPd; 4–SAC305 + CNS3 + AuPd; 5–SAC305 + CNT + AuPd; 6–SAC305 + CNT + Pt

Simultaneously with these processes, the average thickness of the IMC layers at the interface decreased (Fig. 5a). It should be noted that this reduction was experienced in Cu_3_Sn as well as in Cu_6_Sn_5_ layers in all alloys formed by adding the Au–Pd coated CNS1, CNS2, CNS3 and CNTs to the basic SAC305 matrix. The only exception was the solder joint of SAC305 with platinum-coated CNT impurities, where the Cu_6_Sn_5_ IMC layer exhibited a slight increase (Table 2). The average thickness agrees with the values, obtained in our previous studies (Yakymovych et al. 2016; Aspalter et al. 2020). In general, the thickness of the IMC layer can be determined using a simple growth model in which IMC growth is a diffusion-controlled process. Before isothermal aging, the IMC interfacial thickness of the monolithic soldered joint was approximately 10% higher than that of the composite joints. This finding is consistent with statement that the composite solder joints exhibited lower diffusion coefficients, as compared to that of the SAC solder joint (Nai et al. 2009). This signifies that the presence of carbon nanoparticles as reinforcements in the solder joints is effective in retarding the growth of the IMC layer.

**Table 2:** Average thickness (*d*) of the interfacial Cu_3_Sn and Cu_6_Sn_5_ IMCs layers formed at the as-reflow Cu/SAC305 + nanoadmixtures/Cu at 293 K

Alloy	*d*, µm, Cu_3_Sn	*d*, µm, Cu_6_Sn_5_	*d*, µm, Cu_3_Sn + Cu_6_Sn_5_
SAC305	0,7981	4,2697	5,0677
SAC305 + CNS1 + AuPd	0,6563	3,8199	4,4763
SAC305 + CNS2 + AuPd	0,5981	3,9334	4,5315
SAC305 + CNS3 + AuPd	0,6757	3,8974	4,5732
SAC305 + CNT + AuPd	0,5351	3,9334	4,4685
SAC305 + CNT + Pt	0,6337	4,8742	5,5079

Similar phenomena were observed earlier in case of different ceramic admixtures like ZrO_2_ (Gain et al. 2011a) or TiO_2_, (Leong et al. 2011) and were explained on the basis of the adsorption theory of a surface-active material (Tan et al. 2015; Leong et al. 2011; Wang et al. 2015; Gain et al. 2011b). According to this theory, the admixtures of carbon nanoparticles increase the surfactant content of the solder joint and maximizes the number of adsorbed particles on the surface of intermetallic compounds. But at the same time, an increase in the amount of adsorbed material leads to a decrease in the surface energy of IMCs, which, in turn, results in a slowdown in their growth rate both at the interface and in the volume.

**Fig. 6:**
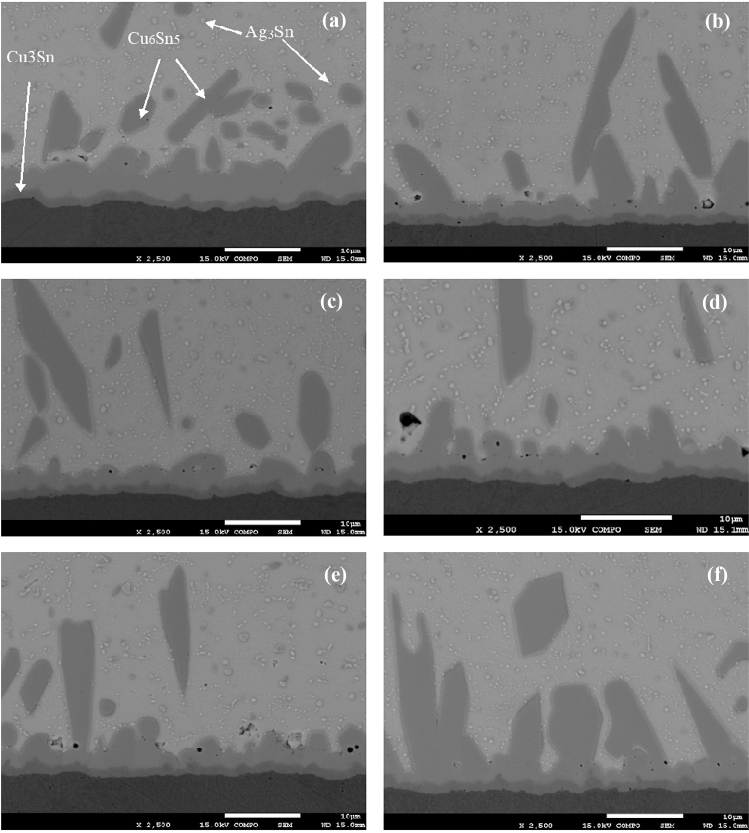
SEM micrographs of SAC305 (**a**), SAC305 + CNS1 + AuPd (**b**); SAC305 + CNS2 + AuPd (**c**); SAC305 + CNS3 + AuPd (**d**); SAC305 + CNT + AuPd (**e**); 6-SAC305 + CNT + Pt (**f**) after aging for 2 months at 253 K

Figure 6 presents the microstructural evolution of the solder joints between the copper substrate and SAC305 matrix armed by CNTs and CNSs nanoadmixtures coated with Au–Pd and Pt, after aging for 2 months at 253 K. As seen from Fig. 6 and Table 3, the morphology of the IMC layers at the solder-substrate interface changed differently in different sample compositions.

**Table 3:** Average thickness (*d*) of the interfacial Cu_3_Sn and Cu_6_Sn_5_ IMCs layers formed at the as-reflow Cu/SAC305 + nanoadmixtures/Cu aged for 2 months at 253 K

Alloy	*d*, µm, Cu_3_Sn	*d*, µm, Cu_6_Sn_5_	*d*, µm, Cu_3_Sn + Cu_6_Sn_5_
SAC305	1,1118	4,9633	6,0750
SAC305 + CNS1 + AuPd	1,1916	4,0512	5,2429
SAC305 + CNS2 + AuPd	1,3384	3,6719	5,0104
SAC305 + CNS3 + AuPd	1,2491	3,4481	4,6972
SAC305 + CNT + AuPd	1,0362	3,7024	4,7386
SAC305 + CNT + Pt	1,3185	5,0669	6,3854

The thickness of the Cu_3_Sn IMC layer increased almost twice on average, namely, from 0.65 µm to 1.21 µm after 2 months aging at 253 K. As for the Cu_6_Sn_5_ layer, its thickness did not change so significantly, except for SAC305 without admixtures, where it increased by an average of 16%. As for other solders with impurities, the average layer thickness in SAC305 + CNS1 + AuPd increased by only 6% and by 4% in SAC305 + CNT + Pt. In the remaining three samples, this thickness even decreased by 6–12%.

Thus, due to slight changes in the thickness of the Cu_6_Sn_5_ layer, the total thickness of the intermetallic layers at the junction of the solder with the copper substrate did not increase significantly. The greatest layer extension was observed in the case of SAC305 without admixtures, while the presence of various nanosized impurities slowed down such a growth (Table 4).

**Table 4:** Percentage change in average thickness (Δ*d*) of the interfacial Cu_3_Sn and Cu_6_Sn_5_ IMCs layers formed at the as-reflow Cu/SAC305 + nanoadmixtures/Cu after aging for 2 months at 253 K

Alloy	Δ*d* %, Cu_3_Sn	Δ*d* %, Cu_6_Sn_5_	Δ*d* %, Cu_3_Sn + Cu_6_Sn_5_
SAC305	39	16	20
SAC305 + CNS1 + AuPd	82	6	17
SAC305 + CNS2 + AuPd	124	– 7	11
SAC305 + CNS3 + AuPd	85	– 12	3
SAC305 + CNT + AuPd	94	-6	6
SAC305 + CNT + Pt	108	4	16

Microstructural analysis of the samples also revealed a noticeable change in the shape of the Cu_6_Sn_5_ formations. As can be seen from Fig. 6, this shape acquires a needle-like structure, and sharp protrusions extending into the metal matrix appear at the solder-substrate interface.

**Fig. 7:**
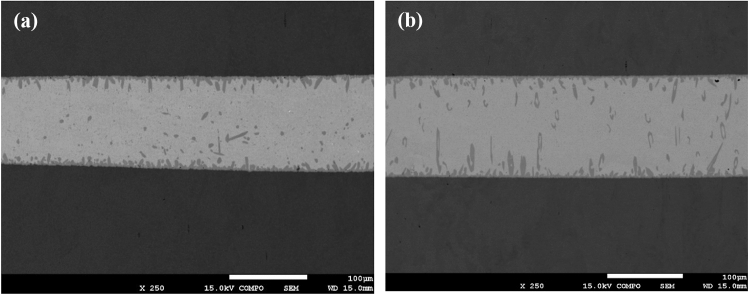
Microstructure of the Cu/SAC305/Cu soldered cross-section before (**a**) and after aging for 2 months at 253 K (**b**)

If we consider the microstructure of not only the areas of the joints adjacent to the substrate, but also the general cross-section of the soldered parts, we can see that appearance of the needle-like formations also occurs in the volume of the SAC matrix (Fig. 7).

**Fig. 8:**
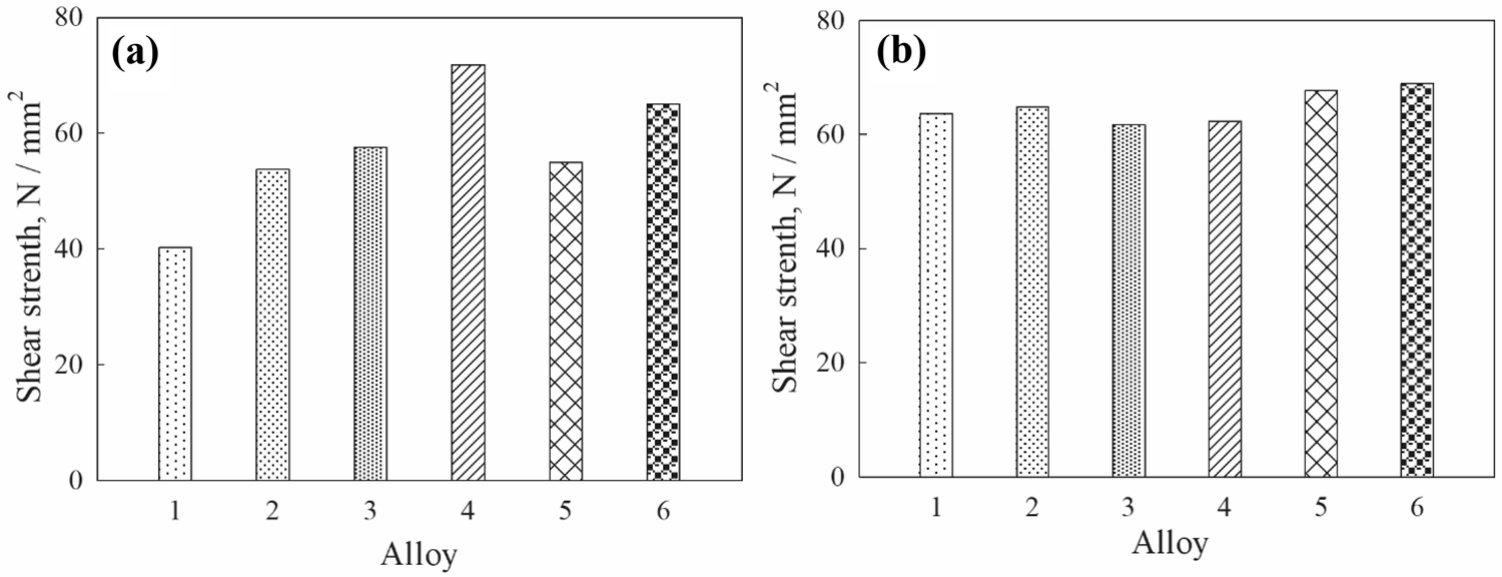
The shear strength of the nanocomposite SAC305 solder joints at 293 K (**a**) and after aging at 253 K (**b**): 1–SAC305, 2–SAC305 + CNS1 + AuPd; 3–SAC305 + CNS2 + AuPd; 4–C305 + CNS3 + AuPd; 5–SAC305 + CNT + AuPd; 6–SAC305 + CNT + Pt

The detected changes in the microstructure of the solder joints also lead to changes in mechanical properties. That is why, shear strength was measured as one of the main properties of connection reliability. As is seen in Fig. 8a, the addition of three types of carbon nanospheres can noticeably increase the shear strength of the SAC305 solder joints, particularly in the case of CNS3 + AuPd. The admixtures of coated carbon nanotubes also increased the shear strength of the joint.

Tensile results after exposure to low temperature (Fig. 8) showed changes in the level of strength in all tested samples.The data obtained are in agreement with findings revealed by Nai, that admixtures of carbon nanotubes to the solder matrix results in increase of the ultimate shear stress and yield stress of the composite solder joints comparing with the unreinforced solder joint (Nai et al. 2008).

It is well known that chemical reactions between the liquid solder and the solid substrate and subsequent diffusion processes in the solid state result in the IMCs growth. The presence of the IMCs between solders and conductor metals is an indication of good metallurgical bonding. A thin, continuous and uniform IMC layer is an essential requirement for good bonding, which increases the shear strength of a solder joint. Up to a certain thickness, such an intermetallic layer can provide a long-term reliable connection of the solder to the substrate, but its excessive growth will deteriorate the joint. However, due to their inherent brittle nature and the tendency to generate structural defects, too thick IMC layer at the solder/substrate interface may degrade the reliability of the solder joints (Frear et al. 1994). Thus, knowledge of the solder/substrate interactions and phase evolution in the solder interconnections is important for the understanding of the reliability of the solder interconnections from the metallurgical viewpoint and for the optimization of the soldering process.

According to the model proposed in (Wu et al. 1993), there are two limiting conditions for intermetallic formation and growth in the solder/copper substrate system. The first is when diffusion through the growing intermetallic layer is the rate-limiting factor (diffusion-controlled growth). The second is when an interfacial reaction is the rate-limiting step (reaction-controlled growth). It was assumed that the intermetallic formation is a thermally activated process dependent on Sn diffusion.

The formation of the intermetallics probably takes place via the following way. Initially after soldering, thin layers of both Cu_6_Sn and Cu_3_Sn are formed at the interface. The η-phase, Cu_6_Sn_5_, forms adjacent to the solder and the ε-phase, Cu_3_Sn, forms adjacent to the Cu substrate. Sn diffuses though the Cu_6_Sn_5_ to the η/ε interface and reacts there with the Cu_3_Sn to form Cu_6_Sn_5_. Sn also diffuses through the Cu_3_Sn phase to the Cu_3_Sn/Cu interface and reacts there with Cu causing the Cu_3_Sn layer to grow in thickness as well. The impurities in composite solders strongly affect the diffusion behavior of Sn and affect the formation of intermetallics at the solder/copper substrate interface, but in different ways. It was established that during annealing of different durations at different elevated temperatures, various admixtures can both increase or decrease the thickness of each of the Cu_3_Sn and Cu_6_Sn_5_ layers, affecting the activation energies of their formation. At the same time, similar studies were not conducted at low sub-zero temperatures. The main result of our research is the conclusion that metal deposited CNTs and CNSs admixtures can slow down the growth of the total amount of intermetallics compared to pure SAC solder. However, the dynamics of the morphology of individual intermetallics requires further research.

However, it should be remembered that adding too many impurities can worsen the mechanical properties of soldered joints. For example, it was reported that the tensile properties improvement for the composites with 0.04 wt.% and 0.07 wt.% of carbon nanotube addition were less significant comparing with 0.01 wt.%. This can be due to the higher level of microporosity found at higher amount of CNTs. The micropores act as centers of stress concentration, which contributed to the formation of microcracks and eventually led to the failure of the material. Besides, the ductility of the composites may decrease with increasing mass percentage of CNTs, as this will occur where the CNTs will be in contact with each other and not with the solder particles. This will result in the formation of small clusters, which will prevent effective bonding between carbon nanotubes and solder particles. Thus, small cracks can appear, which act as nucleation centers of plastic instability, leading to the failure of the material with lower values of plasticity. We assume that the same behavior is possible in the case of carbon nanospheres, which addition to solder matrix has practically not been investigated before. It is possible that the properties of CNSs obtained by different methods are slightly different, and therefore their optimal proportions in the solder should also be different, which concerns, in particular, the shear strength reduction in case of the SAC305 + CNS3 + AuPd alloy.

Finally, it should be noted that the shear strength of soldered joints, like some other mechanical properties, may depend not only on the intermetallic layers formed at the solder/substrate interface, but also on the amount, size and morphology of the intermetallics formed in the solder bulk. At this point, further studies are required.

## Conclusions

As a result of prolonged exposure at a low temperature of 253 K, the thickness of the intermetallic layers formed in copper pairs soldered with SAC305 nanocomposite solders containing carbon nanoparticles coated by Au–Pd and Pt increases more slowly than in the case of solders without impurities. The addition of coated carbon nanospheres and nanotubes increased the shear strength of the joints.

There is a noticeable change in the shape of the Cu_6_Sn_5_ intermetallic areas, which acquire a needle-like structure. Sharp protrusions directed towards the middle of the metal matrix appear at the solder-substrate interface. Needle-like formations are also formed in the volume of the SAC matrix, mainly in the direction perpendicular to the copper substrates.

Thus, the use of the nanocomposite SAC305 solders in electronic devices operating at temperatures as low as –20 ℃ degrees is possible at least up to 2 months, but it should be taken into account that further growth of intermetallic layers up to the dimensions of the joint width between the soldered parts can cause the change of solder structure from ductile to quasi-ductile and later to quasi-brittle one, until the brittle fracture mode is attained.

## Abbreviations

LFS: Lead-free solders
SAC305: Sn3.0Ag0.5Cu
CNT: Carbon nanotubes
CNS: Carbon nanospheres
SEM: Scanning electron microscope
TEM: Transmission electron microscope
